# Female vs. male relative fatality risk in fatal motor vehicle crashes in the US, 1975–2020

**DOI:** 10.1371/journal.pone.0297211

**Published:** 2024-02-12

**Authors:** Mitchell Z. Abrams, Cameron R. Bass

**Affiliations:** 1 Department of Biomedical Engineering, Injury and Orthopedic Biomechanics Laboratory, Duke University, Durham, North Carolina, United States of America; 2 Department of Biomedical Engineering, Wayne State University, Detroit, Michigan, United States of America; Tsinghua University, CHINA

## Abstract

Motor vehicle accidents are the leading cause of death for young adults 18–29 years old worldwide, resulting in nearly 1 million years of life lost annually in the United States. Despite improvements in vehicle safety technologies, young women are at higher risk of dying in car crashes compared with men in matched scenarios. Vehicle crash testing primarily revolves around test dummies representative of the 50th percentile adult male, potentially resulting in these differences in fatality risk for female occupants compared to males. Vehicle occupants involved in fatal car crashes were matched using seating location, vehicle type, airbag deployment, seatbelt usage, and age. The relative risk for fatality (R) between males and females was calculated using a Double Pair Comparison. Young women (20s-40s) are at approximately 20% higher risk of dying in car crashes compared with men of the same age in matched scenarios. In passenger cars, 25-year-old female occupants in passenger car crashes from 1975–2020 exhibit R = 1.201 (95% CI 1.160–1.250) compared to 25-year-old males, and R-1.117 (95% CI 1.040–1.207) for passenger car crashes from 2010–2020. This trend persists across vehicle type, airbag deployment, seatbelt use, and number of vehicles involved in a crash. Known sex-based differences do not explain this large risk differential, suggesting a need for expanded test methodologies and research strategies to address as-yet unexplored sex differences in crash fatalities. These differences should be further investigated to ensure equitable crash protection.

## Introduction

Motor vehicle accidents are one of the leading causes of mortality worldwide, and the leading cause of death for young adults 18–29 years old [1]. In the United States, mortality from motor vehicle accidents is the 3^rd^ leading nationwide cause of unintentional injury death, resulting in over 900,000 years of life lost annually as of 2020 [2]. Motor vehicle fatalities rank in the top 3 causes of death for individuals under the age of 34 [3].

**Fig 1:**
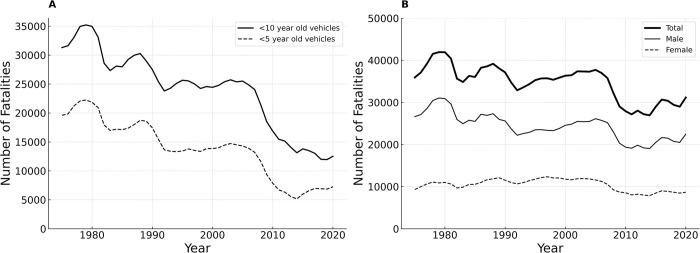
Trends in annual vehicle fatality rates. **(A)** Annual vehicle occupant fatalities involving vehicles manufactured in the prior five or 10 years. Annual fatalities for occupants in “recent-model” vehicles manufactured in the last five or 10 years have been generally declining as vehicle safety technologies have improved. **(B)** Annual vehicle occupant fatalities have decreased overall since 1975, yet fatalities among females have remained largely the same year-to-year.

The National Highway Traffic Safety Administration (NHTSA) maintains the Fatality Analysis Reporting System (FARS) [4,5], which tracks all traffic crashes in the USA since 1975 that involve at least one fatality. FARS data are used to inform safety decisions at the local, state, and national levels, and provide key insights into the efficacy of changing vehicle and roadway safety standards [4]. To be included in FARS, a crash must occur on a public road and must result in at least one death within 30 days of the crash. Road fatalities in the US continue to decrease as better advanced safety technologies emerge and become standard features across the board. Occupant fatalities involving vehicles manufactured in the last five or ten years have decreased steadily, down significantly since 1975 (Fig 1A). Despite improvements, when compared to 15 peer nations, the United States ranks last in reducing the rate of annual vehicle fatalities, and fatalities in 2021 were the highest since 2005 [6,7].

Overall, evolving crash testing and vehicle standards are estimated to have prevented ~60% of all potential vehicle fatalities in the USA [8,9]. However, the bulk of the fatality reduction is in males (Fig 1B). Qualitatively, female fatality rates have been relatively stable when compared to the overall trend. While historically males have driven more miles per licensed driver than females, that gap is steadily closing, with decreasing differences in licensure rates and driving exposure [10–12]. Driving exposure for females has increased, and female drivers have been reported to display similar behavioral risk profiles to males when behind the wheel [12–14].

Given these trends, it is important to note that no vehicle crashworthiness test in the US involves an anthropomorphic test device (ATD) representative of the average adult female, despite clear evidence of the increase of female drivers over the last 20 years [15]. The current US regulations only require crash testing with the 50^th^ percentile adult male and 5^th^ percentile adult female ATD. Prior research has shown that female drivers and vehicle occupants are more likely than males to suffer severe or fatal injuries when involved in a crash severe enough to result in fatality for an occupant involved in the crash [14,16–18]. Female vehicle occupants are at higher risk of Abbreviated Injury Scale (AIS) 2+ (moderate) and 3+ (serious) injuries compared to males when controlling for a variety of factors, particularly to the lower extremities [19,20]. This disparity between men and women in automobile crashes is a major public health issue with implications for automobile design, personnel protection, and governmental regulation. The goal of this paper is to utilize fatal crash data to assess the extent to which a difference in fatality risk exists for male and female occupants under matched crash conditions.

## Materials and methods

### Data pre-processing

Data were downloaded from the US Department of Transportation–National Highway Traffic Safety Administration Fatality Analysis Reporting System (NHTSA FARS) FTP directory. Data pre-processing was performed using the fars-cleaner package, produced by the authors for this study and freely available as open source software [21,22]. Pre-processing with this package merged changes in the FARS dataset, adjusting outdated and modified codes using the FARS Analytical User Manual as reference [4]. Data preprocessing and analyses were performed using Python (RRID:SCR_008394), pyjanitor, pandas dataframes (RRID:SCR_018214) and Jupyter notebooks (RRID:SCR_018315) parameterized with Papermill [23–27]. Data visualizations were produced with matplotlib (RRID:SCR_008624) [28].

### Double pair comparison method

The double pair comparison method developed by Evans isolates specific features of fatality risk in a crash on a comparable basis [29]. One group of vehicle occupants is selected as the subject occupants, and another is selected as the control. The assessment of fatality risk is performed across the control group. To illustrate the use of the double pair method to determine the relative fatality risk of female vs. male drivers, two sets of crashes are selected with a consistent control occupant (for example, male passengers seated in the front right seat of the vehicle) so that

A=Numberoffemaledriverskilledinvehicleswithacontroloccupant. (1)

B=Numberofcontroloccupantskilledinvehicleswithafemaledriver. (2)

C=Numberofmaledriverskilledinvehicleswithacontroloccupant. (3)

D=Numberofcontroloccupantskilledinvehicleswithamaledriver. (4)

From these counts, the relative risk of fatality for a female driver vs. the control occupant, *r*_1_ = *A*/*B*, and the relative risk of fatality for a male driver vs. the control occupant, *r*_2_ =*C*/*D*, were used to derive the female vs. male relative risk, *R* = *r*_1_/*r*_2_. In this technique, the control occupants (B and D) are eliminated from the calculation of relative risk, so male and female subject occupants can be compared. The original double pair method determines standard error in the estimates of *R* (Δ*R*):

$$\Delta R=R\sqrt{\sigma_{\mu }^{2}+\frac{1}{A}+\frac{1}{B}+\frac{1}{C}+\frac{1}{D}}$$ (5)

where *σ*_*μ*_ is an estimate of “intrinsic uncertainty,” set to either 0.05 or 0.1 [17,29]. The introduction of this constant term forces all variance estimates to be similar, regardless of the pointwise variance in each estimate. Since these are used in the weighted summaries, results may be biased by granting larger weight to samples with more uncertainty. To mitigate this bias, an alternative method was used for describing the variance of the risk ratios [30]. To correct this deficiency, the counts were further stratified using:

A=Numberoffemaledriverskilledinvehicleswithacontroloccupant(alsokilled). (6)

B=Numberoffemaledriverskilledinvehicleswithacontroloccupant(notkilled). (7)

C=Numberofcontroloccupantskilledinvehicleswithafemaledriver(notkilled). (8)

E=Numberofmaledriverskilledinvehicleswithacontroloccupant(alsokilled). (9)

F=Numberofmaledriverskilledinvehicleswithacontroloccupant(notkilled). (10)

G=Numberofcontroloccupantskilledinvehicleswithamaledriver(notkilled). (11)

Note that the original variables **A**, **B**, **C**, and **D** (Eqs 1–4) can be derived as the sum of the new counts detailed above (Eqs 6–11). Now, the relative risk ratio is

R=A+BA+CE+FE+G. (12)

The variance for the log of the relative risk ratio is [30]

$$\Delta R=\frac{\left[\left(A\times (A+B+C)+(B\times C))\times (F+G\right))+[\left(E\times (E+F+G)+(F\times G))\times (B+C\right)\right]}{\left(A+B)\times (A+C)\times (E+F)\times (E+G\right)}$$ (13)

Weighted risk ratios ($\bar{R}$) and weighted estimates of variance ($\Delta \bar{R}$) are

$$\bar{R}=\exp\left(\frac{\sum \left(\ln R\times 1/\Delta R\right)}{\sum 1/\Delta R}\right)$$ (14)

$$\Delta \bar{R}=\frac{1}{\sum 1/\Delta R}.$$ (15)

Confidence intervals (95%) are derived with a bootstrap method, sampling with replacement many times and calculating values for $\bar{R}$ and $\Delta \bar{R}$ for each new sample set [30]. This procedure was replicated 5000 times for each weighted average. Each bootstrap run produces a distribution of results, from which the 95% confidence interval is derived.

Cases were selected with at least two occupants and with at least one fatality in the vehicle. All rollover crashes were excluded. Only fatalities within a passenger car were examined. Fatality was determined as coded within FARS and includes those declared dead at the crash and within 30 days from crash-related causes. Age ranges were examined in five-year periods for subject occupants, while control occupants were grouped as in previous analyses: ages 16–24, 25–34, 35–54, and 55+ [17]. Tables A and B in S1 Appendix show the subject breakdown for 1975–2020 with matched airbag deployment in passenger cars (*n* = 249,160 fatalities) and light trucks (*n* = 92,826 fatalities) and 2010–2020 with matched airbag deployment in passenger cars (*n =* 26,221 fatalities). Matched airbag deployment was defined as cases where both the subject and the control occupant experienced the same airbag deployment at their seating position (deployment or no deployment). Cases with unknown airbag deployment were excluded.

Fatal crash cases were grouped by subject occupant seating position (driver seat, front right seat, rear left seat, etc.), seat-belt use, and control occupant seating position and age. In each analysis, a weighted average of the value for *R* was taken within each subject age subset to find the overall risk a subject of that age independent of seating position or control occupant.

Second row passengers were included as subjects for analyses spanning 1975–2020 only. From 2010–2020, there were typically fewer than 15 fatalities in each subset (age, seating position, belt use, airbag) for rear passengers, and in most cases there were 1 or 0 in each subset. This resulted in excessive error due to small *n*, so rear passengers were not considered from 2010–2020.

## Results

**Fig 2:**
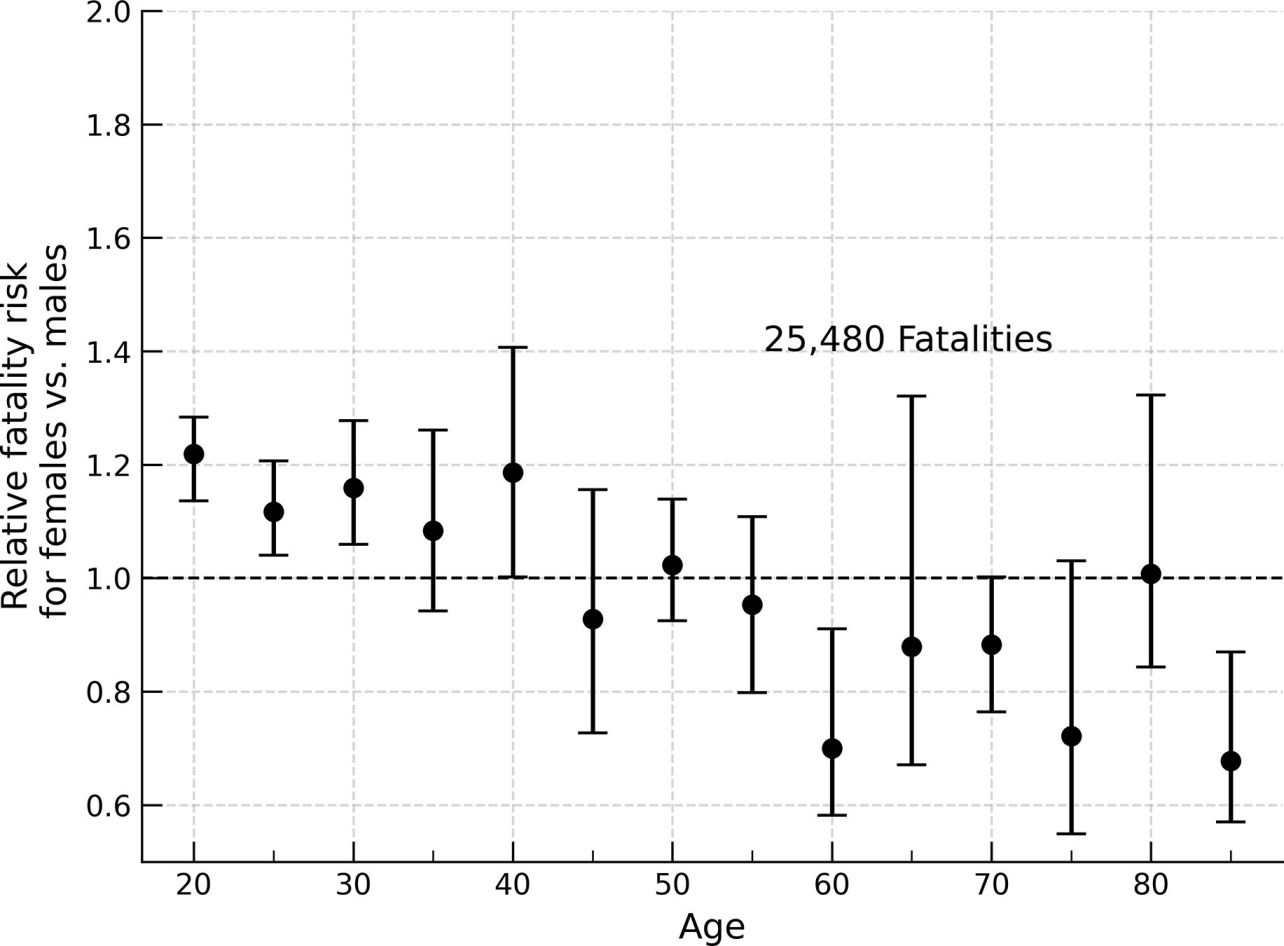
Relative fatality risk, females vs. males, passenger car fatalities 2010–2020. Relative fatality risk for all female occupants compared to males (*n* = 25,480; 2010–2020) under matched airbag deployment condition. Young female vehicle occupants are at an increased risk of fatality compared to males until ~40 years old.

**Fig 3:**
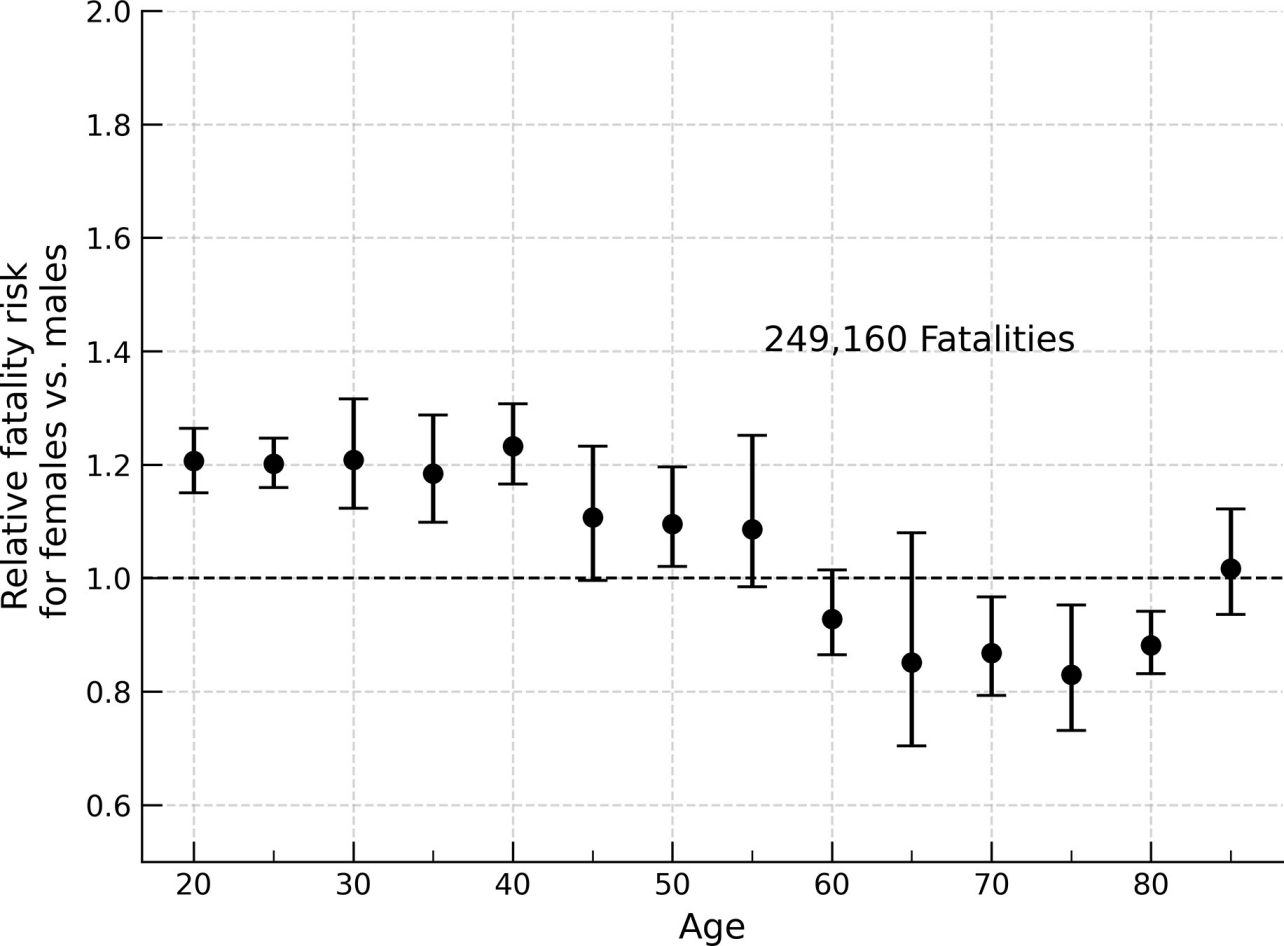
Relative fatality risk, females vs. males, passenger car fatalities 1975–2020. Relative fatality risk for female occupants compared to males (*n* = 249,160; 1975–2020) under matched airbag deployment condition. Young female vehicle occupants (driver, front right passenger, left and right second row passengers) in passenger cars are at an approximately 20% increased risk of fatality compared to males until ~40 years old.

Female vehicle occupants between ages 20 and 40 are ~20% more likely to die in a crash than males of the same age under matched conditions (Figs 2 and 3). This general trend is apparent when isolating vehicle type (passenger car vs. light trucks), seat-belt use (belted vs unbelted), number of vehicles involved (one, two, or three or more vehicles), crash direction (front vs side), and airbag deployment, and persists for females up to age 50. To best illustrate the results produced by the double pair method, consider the case of belted drivers, aged 23–27 years (25-year-old drivers), in passenger cars, using belted front-right seat passengers as a control, matching airbag deployment, in crashes between 2010 and 2020. These results are given in Table 1 for each sex/age cohort under these conditions, giving a relative risk of 4.7% higher for 25 year old, belted female drivers with matched airbag deployment compared to 25 year old male drivers under the same conditions (Relative Risk (R) = 1.047; 95% Confidence Interval (CI) = 0.836–1.168).

**Table 1:** Female vs. Male Fatality Risk, Belted 25YO Car Drivers, airbag deployment (Matched), 2010–2020.

Control Occupant Characteristics, Age	Fatalities				Ratios		
	A	B	C	D	r_1_ = A/B	r_2_ = C/D	R = *r*_*1*_/*r*_*2*_
Male Passenger, 16–24yo	27	34	158	139	0.794	1.137	0.699
Male Passenger, 25–34yo	59	53	103	110	1.113	0.936	1.189
Male Passenger, 35–54yo	16	16	24	37	1.0	0.649	1.542
Male Passenger, 55+yo	1	7	3	8	0.143	0.375	0.381
Female Passenger, 16–24yo	35	29	133	140	1.207	0.95	1.27
Female Passenger, 25–34yo	33	41	74	94	0.805	0.787	1.022
Female Passenger, 35–54yo	13	30	12	23	0.433	0.522	0.831
Female Passenger, 55+yo	3	40	1	26	0.075	0.038	1.95
Weighted average R [95% confidence]							1.047 [0.832, 1.267]

Control occupants are belted, front-right passengers.

Matched airbag deployment refers to cases where the airbag either deploys at both the control and subject occupant seating position, or deploys at neither position.

**Fig 4:**
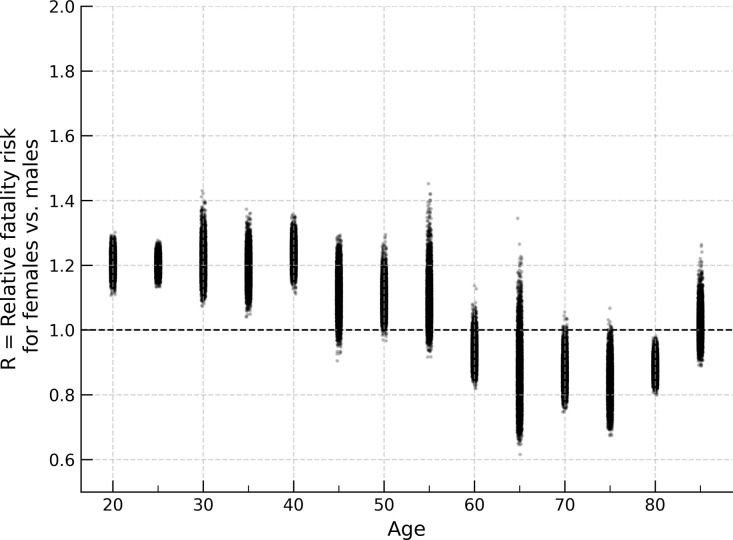
Bootstrap estimates, females vs. males, passenger car fatalities 1975–2020. Bootstrap estimates for relative fatality risk for female occupants compared to males under matched airbag deployment condition. 95% confidence intervals are derived from the middle 95% of these estimates.

Taking the weighted average across the controls presented in Table 1 for each set of subject occupants, we produce Table 2, for 25 year old vehicle occupants. Across cases for these occupants, the overall risk (Table 2) is 11.7% (R = 1.117, 95% CI 1.040–1.207) higher risk for females. This value is plotted as the corresponding value in Fig 2. Repeating this analysis for female occupants at other ages, we find younger females have a higher fatality risk than younger males when driving or sitting in the front passenger seat, regardless of seat-belt use (Table 2). Fig 3 shows the results of repeating this analysis for all crashes from 1975 to 2020. Fig 4 is representative of the confidence interval estimation utilizing the bootstrap method, and shows the distribution of R values.

**Table 2:** Values of R, fatality risk to 25yo females compared to 25yo males, passenger car occupants, 2010–2020, matched airbag deployment.

Subject Occupant	Female Fatalities	Male Fatalities	Total Fatalities	R	95% CI
Unbelted drivers, airbag deployed	114	427	541	0.987	[0.916, 1.05]
Unbelted right front passengers, airbag deployed	225	346	571	1.297	[1.050, 1.511]
Belted drivers, airbag deployed	187	508	695	1.047	[0.832, 1.267]
Belted right front passengers, airbag deployed	352	385	737	1.143	[0.943, 1.374]
Unbelted drivers, airbag not deployed	78	226	304	1.255	[1.033, 1.856]
Unbelted right front passengers, airbag not deployed	87	170	257	0.978	[0.656, 1.439]
Belted drivers, airbag not deployed	70	196	266	1.292	[1.003, 2.410]
Belted right front passengers, airbag not deployed	101	163	264	1.095	[0.789, 1.350]
**Weighted Average**				1.117	[1.040, 1.207]

**Fig 5:**
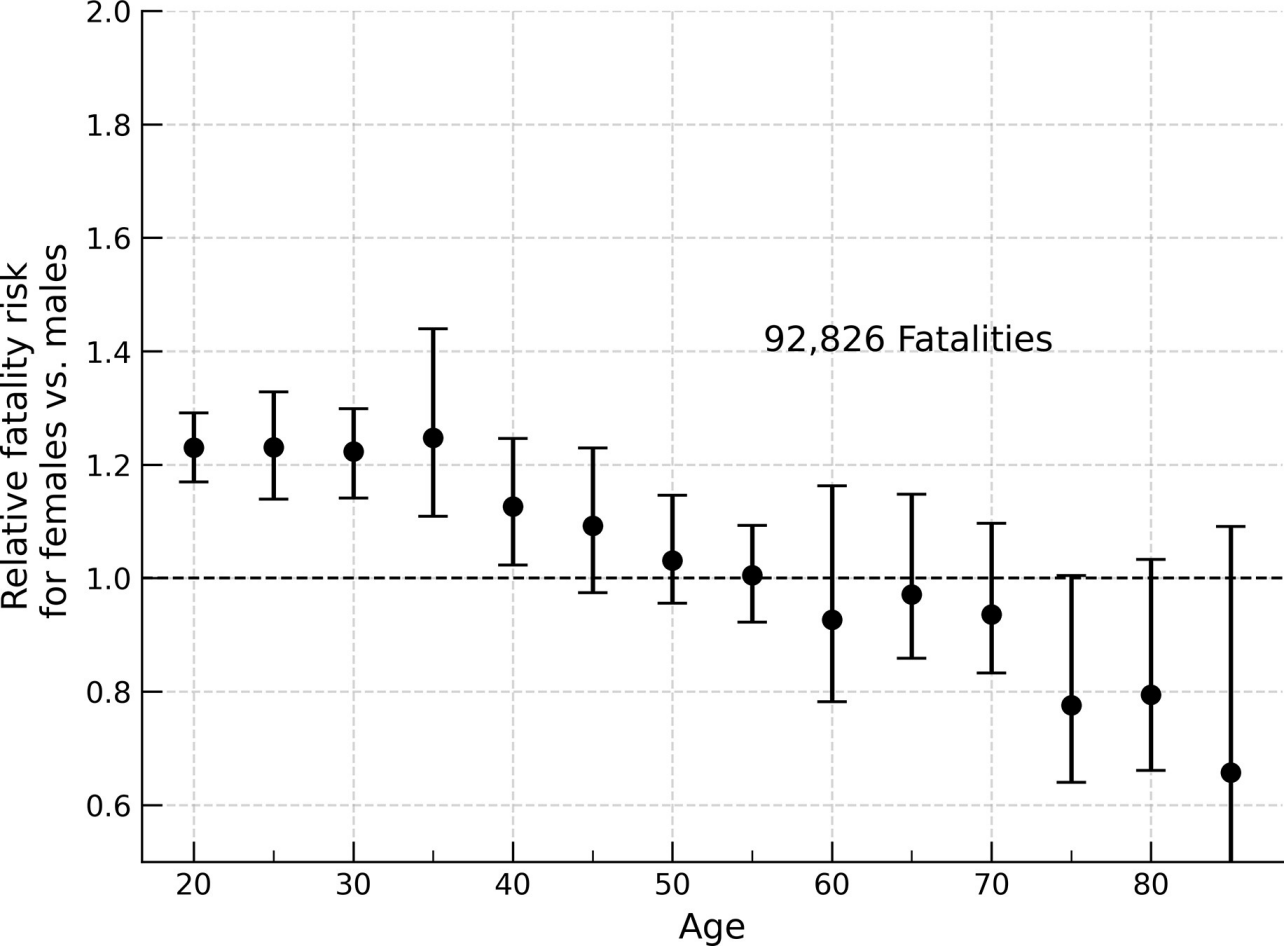
Relative fatality risk, females vs. males, light truck fatalities 1975–2020. Relative fatality risk for female occupants in light trucks compared to males (*n* = 92,826; 1975–2020) under matched airbag deployment condition. Young female vehicle occupants in light trucks (driver, front right passenger, left and right second row passenger) are at an approximately 20% increased risk of fatality compared to males until ~40 years old.

Repeating the analysis for light truck occupants, we find a similar trend as in passenger cars, as shown in Fig 5. Female occupants in passenger cars and light trucks are at increased risk to males in one and two vehicle crashes (Fig A-D in S1 Appendix). There is no difference in fatality risk for females compared to males in crashes involving three or more vehicles (Fig E and Fig F in S1 Appendix). Females are at increased risk in frontal impacts and passenger side impacts, but not in driver side impacts (Fig G-I in S1 Appendix).

## Discussion

Despite significant advances in vehicle safety since 1975, female vehicle occupants involved in fatal crashes have a higher risk of death compared to males in matched circumstances. These results show that when in a fatal crash, a younger female occupant is approximately 20% more likely to suffer a fatal injury than a male occupant of the same age, regardless of seating position, airbag deployment, or seat-belt usage.

We have investigated several potential covariates that might explain these findings, including number of passengers, number of vehicles involved, and drug/alcohol use. There are no significant differences in the number of occupants in a vehicle between male and female drivers (Mann-Whitney U-test, p>0.90; See Fig J in S1 Appendix). While there are slight differences in the proportion of male and female fatalities identified as using drugs or alcohol, the magnitude of these differences is insufficient to account for the 20% increase in risk shown by young female occupants.

The distribution of vehicles driven by females is similar to that for males, with a slightly higher proportion of female drivers using cars with vehicle masses of ~2,500 lb, but both sexes use heavier vehicles at the same rate. While male drivers are more frequently involved in single-car crashes compared to female drivers, the proportion of crashes involving multiple vehicles is similar across sex. The distribution by sex of the number of occupants in a vehicle are similar as well. The lack of a large qualitative difference in these covariates would imply limited effect on the relative risk to drivers. To verify this conclusion, the above analyses were applied to subsets of vehicle occupants involved in one-, two- and multi-vehicle crashes, as well as cases with one, two, or several vehicle occupants.

When examining single vehicle crashes, we continue to find a higher relative risk to female occupants compared to males despite the increased proportion of crashes involving males. This is true for both passenger cars and light trucks. Similarly, there is an increased risk to female occupants in crashes with two vehicles when compared to males, but not in crashes with three or more vehicles. While females are not at higher risk than males in crashes with 3+ vehicles, one- and two-vehicle crashes account for the majority of cases in the FARS dataset (Fig K in S1 Appendix).

The results presented here are similar to those presented by Evans in 2001 for FARS data through 1998 [17]. Notably, the inclusion of data from before 2010 in the analysis presented in Fig 3 mainly reduces the error assessment, without qualitatively altering the overall characteristics of the risk curve. While analyses from 1975–2020 include second-row passengers, we excluded this subject class from analyses for 2010–2020 due to small *n*. The small group size (total *n* = 662 for belted and unbelted male and female rear passengers) resulted in large subgroup error estimates, which overly influence the error estimates. This limits the generalizability of the results for recent crash cases. However, the overall trend is apparent for all analyses with substantial group sizes. As overall there are more male fatalities per year than female fatalities, a sufficiently large sample size may be necessary to observe the differential risk presented here.

Physiological/anatomical differences are difficult to explore within the FARS dataset, but may provide an explanation. Current vehicle crashworthiness tests do not use a crash dummy representative of the average adult female [15]. The current US regulations require crash testing primarily with the 50^th^ percentile Hybrid III (HIII) adult male ATD, with a few tests adding in the 5^th^ percentile adult female as a passenger. Since the 5^th^ percentile female ATD is primarily a dimensionally scaled version of the HIII male [31], the physiological and anatomical differences between the sexes may not be completely reproduced in testing methods. For example, a recent study identified differences in positioning female cadaver specimens compared to male specimens during reclined frontal impacts, requiring a forward shift of the belt anchor points to produce comparable kinematics and injuries for specimens of both sexes [32].

These results are not completely consistent with other well-described trends related to sex differences in injury. Females are more likely than males to suffer fractures past the age of 60 due to osteoporosis and to experience bone loss at an earlier age [33], and females tend to have greater age related bone density loss than males [34]. Increased fracture risk in elderly females, therefore, cannot explain the observed trends. Without detailed injury report data (not available within FARS), the cause of death cannot be determined for each case. This information would be valuable in parsing the differences between male and female crash survivability. We posit that there may be unobserved trends in the injury patterns, and therefore outcomes, between male and female occupants. These trends may be the result of unintentional vehicle design issues, or a potentially unexplained lack of biofidelity in the ATDs used in testing.

## Supporting information

S1 Appendix The supplemental material contains supporting tables (S1 Appendix Table A-D and S1 Appendix Fig A-K). (DOCX)
