# Supplementary material for: Female vs. male relative fatality risk in fatal motor vehicle crashes in the US, 1975–2020
Source: PLoS One. 2024 Feb 12;19(2):e0297211. doi: 10.1371/journal.pone.0297211 (PMC10861033; doi:10.1371/journal.pone.0297211)

**S1 Appendix. Distribution of occupants in each analysis.**

**Table A. Distribution of 249,160 fatally injured subject occupants, 1975-2020, matched airbag condition, passenger cars.**

| **Vehicle** | **Subject Occupant** | **Female Fatalities** | **Male Fatalities** | **Total** |
| --- | --- | --- | --- | --- |
| Car | Unbelted drivers, airbag deployed | 1,419 | 4,176 | 5,595 |
| Car | Unbelted right front passengers, airbag deployed | 2508 | 3284 | 5,792 |
| Car | Belted drivers, airbag deployed | 4,160 | 7,595 | 11,755 |
| Car | Belted right front passengers, airbag deployed | 7,881 | 4,922 | 12,803 |
| Car | Unbelted drivers, airbag not deployed | 17,008 | 54,148 | 71,156 |
| Car | Unbelted right front passengers, airbag not deployed | 34,638 | 39,076 | 73,714 |
| Car | Belted drivers, airbag not deployed | 8,072 | 15,301 | 23,373 |
| Car | Belted right front passengers, airbag not deployed | 17,373 | 9,815 | 27,188 |
| Car | Unbelted rear passengers, airbag not deployed | 5,543 | 9,031 | 14,574 |
| Car | Belted rear passengers, airbag not deployed | 1,924 | 1,286 | 3,210 |
| **Totals** |  | **100,526** | **148,634** | **249,160** |

**Table B. Distribution of 92,826 fatally injured subject occupants, 1975-2020, matched airbag condition, light trucks.**

| **Vehicle** | **Subject Occupant** | **Female Fatalities** | **Male Fatalities** | **Total** |
| --- | --- | --- | --- | --- |
| Light truck | Unbelted drivers, airbag deployed | 502 | 1,979 | 2,481 |
| Light truck | Unbelted right front passengers, airbag deployed | 1,027 | 1,370 | 2,397 |
| Light truck | Belted drivers, airbag deployed | 1,470 | 3,715 | 5,185 |
| Light truck | Belted right front passengers, airbag deployed | 3,217 | 1,891 | 5,108 |
| Light truck | Unbelted drivers, airbag not deployed | 4,059 | 25,145 | 29,204 |
| Light truck | Unbelted right front passengers, airbag not deployed | 9,947 | 17,387 | 27,334 |
| Light truck | Belted drivers, airbag not deployed | 2,145 | 6,529 | 8,674 |
| Light truck | Belted right front passengers, airbag not deployed | 4,849 | 3,825 | 8,674 |
| Light truck | Unbelted rear passengers, airbag not deployed | 1,042 | 1,689 | 2,731 |
| Light truck | Belted rear passengers, airbag not deployed | 610 | 428 | 1,038 |
| **Totals** |  | **28,868** | **63,958** | **92,826** |

**Table C. Distribution of 25,480 fatally injured subject occupants, passenger cars 2010-2020, matched airbag condition.**

| **Vehicle** | **Subject Occupant** | **Female Fatalities** | **Male Fatalities** | **Total** |
| --- | --- | --- | --- | --- |
| Car | Unbelted drivers, airbag deployed | 649 | 1,838 | 2,487 |
| Car | Unbelted right front passengers, airbag deployed | 1,162 | 1,380 | 2,542 |
| Car | Belted drivers, airbag deployed | 2,132 | 3,987 | 6,119 |
| Car | Belted right front passengers, airbag deployed | 4,137 | 2,610 | 6,747 |
| Car | Unbelted drivers, airbag not deployed | 346 | 1,006 | 1,352 |
| Car | Unbelted right front passengers, airbag not deployed | 553 | 757 | 1,310 |
| Car | Belted drivers, airbag not deployed | 731 | 1,646 | 2,377 |
| Car | Belted right front passengers, airbag not deployed | 1,440 | 1,106 | 2,546 |
| **Totals** |  | **11,150** | **14,330** | **25,480** |

**Table D. Distribution of 26,221 fatally injured subject occupants, passenger cars, 2010-2020, matched airbag condition.**

| **Vehicle** | **Subject Occupant** | **Female Fatalities** | **Male Fatalities** | **Total** |
| --- | --- | --- | --- | --- |
| Car | Unbelted drivers, airbag deployed | 651 | 1,848 | 2,499 |
| Car | Unbelted right front passengers, airbag deployed | 1,168 | 1,385 | 2,553 |
| Car | Belted drivers, airbag deployed | 2,164 | 4,112 | 6,276 |
| Car | Belted right front passengers, airbag deployed | 4,400 | 2,707 | 7,107 |
| Car | Unbelted drivers, airbag not deployed | 346 | 1,006 | 1,352 |
| Car | Unbelted right front passengers, airbag not deployed | 557 | 758 | 1,315 |
| Car | Belted drivers, airbag not deployed | 747 | 1,696 | 2,443 |
| Car | Belted right front passengers, airbag not deployed | 1,523 | 1,153 | 2,676 |
| Car | Unbelted rear passengers, airbag not deployed | 123 | 254 | 377 |
| Car | Belted rear passengers, airbag not deployed | 145 | 148 | 293 |
| **Totals** |  | **11,824** | **15,067** | **26,891** |

**Figure A. Relative fatality risk, females vs. males, passenger car fatalities, single car crashes, 1975-2020.**

**
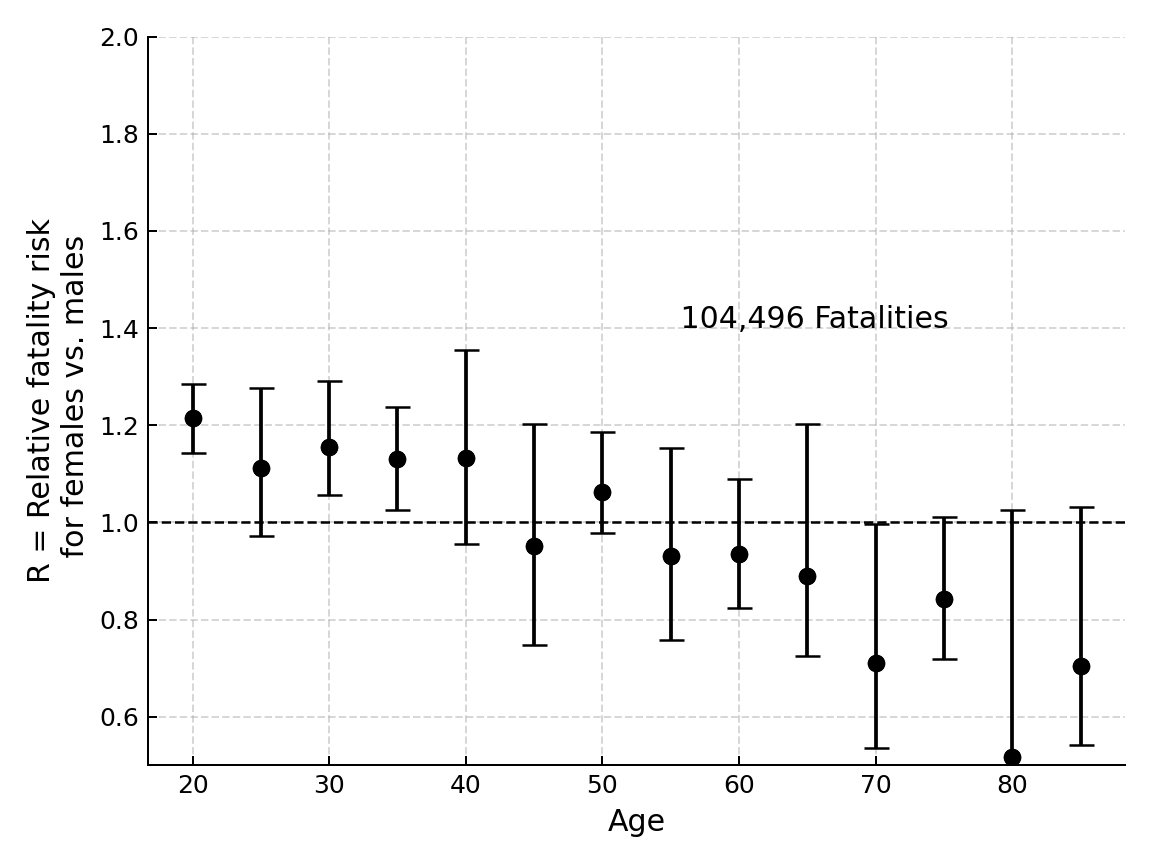
**

**Figure B. Relative fatality risk, females vs. males, light truck fatalities, single car crashes, 1975-2020.**

**
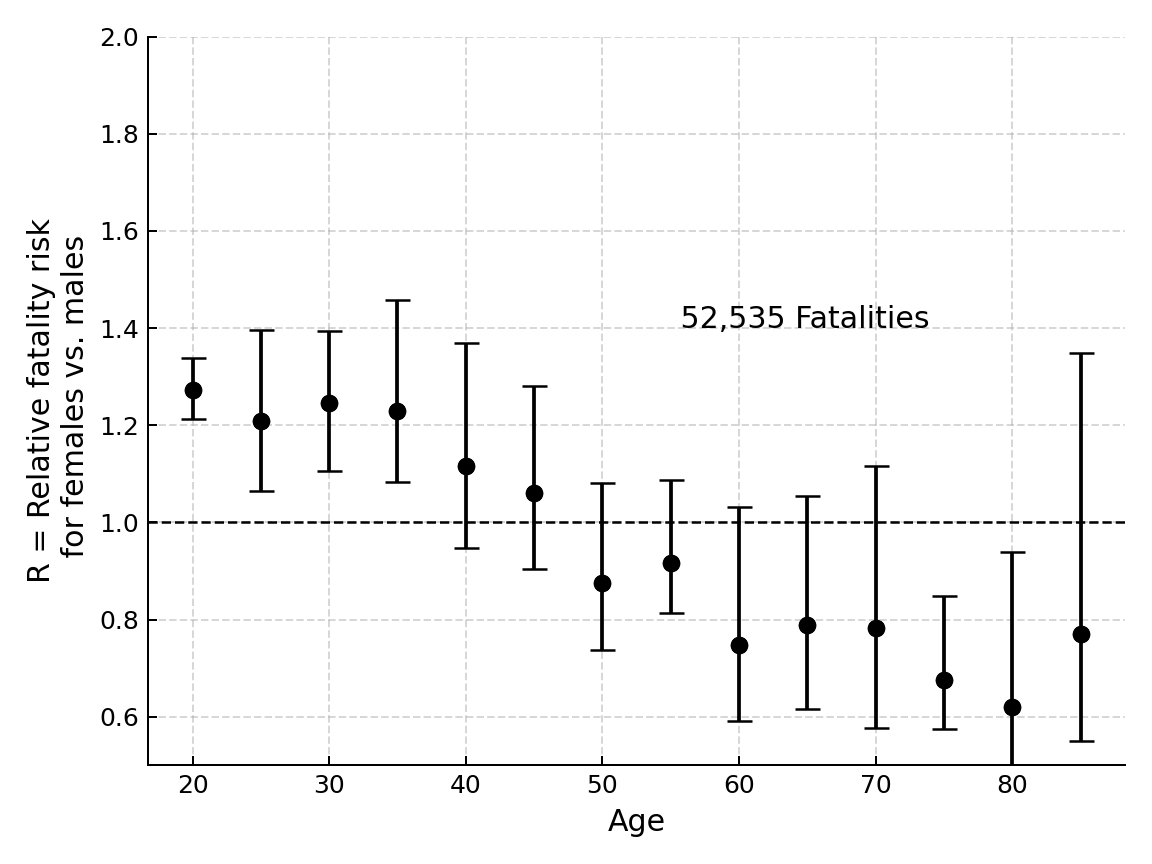
**

**Figure C. Relative fatality risk, females vs. males, passenger car fatalities, two car crashes, 1975-2020.**

**
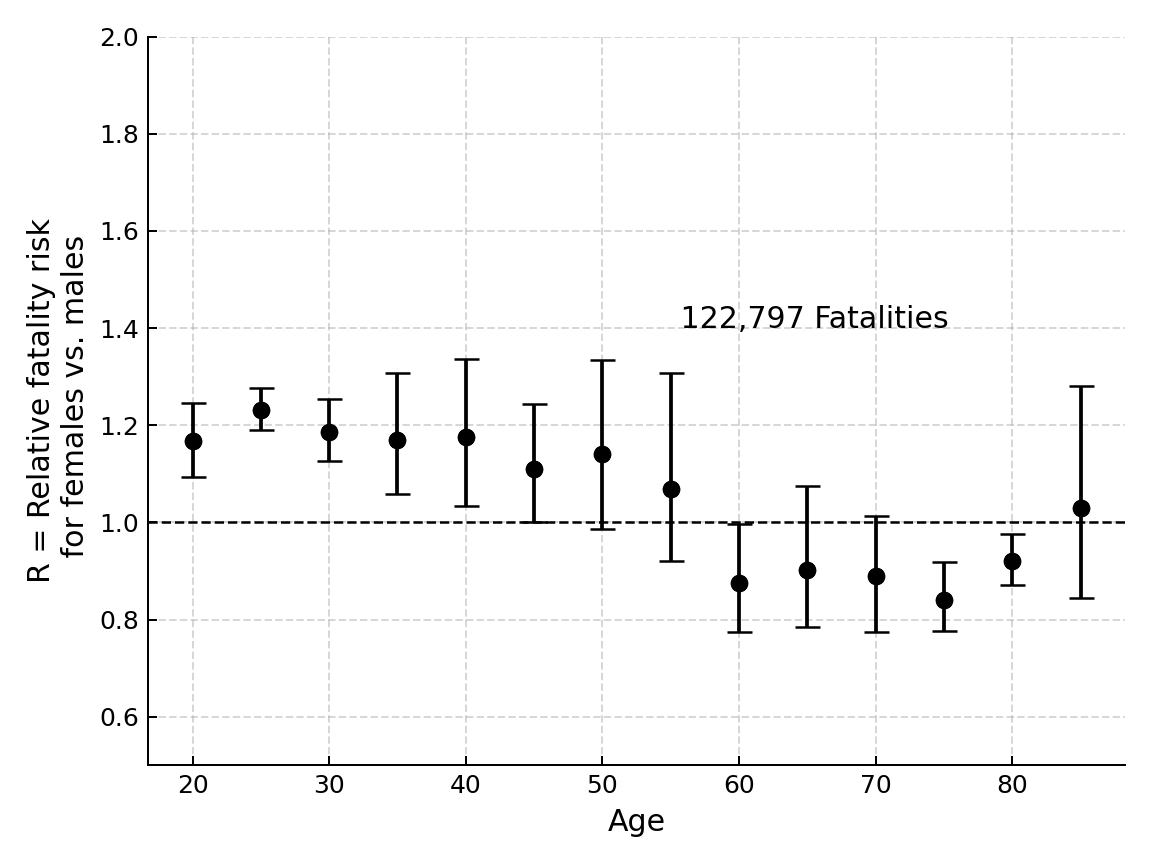
**

**Figure D. Relative fatality risk, females vs. males, light truck fatalities, two car crashes, 1975-2020.**


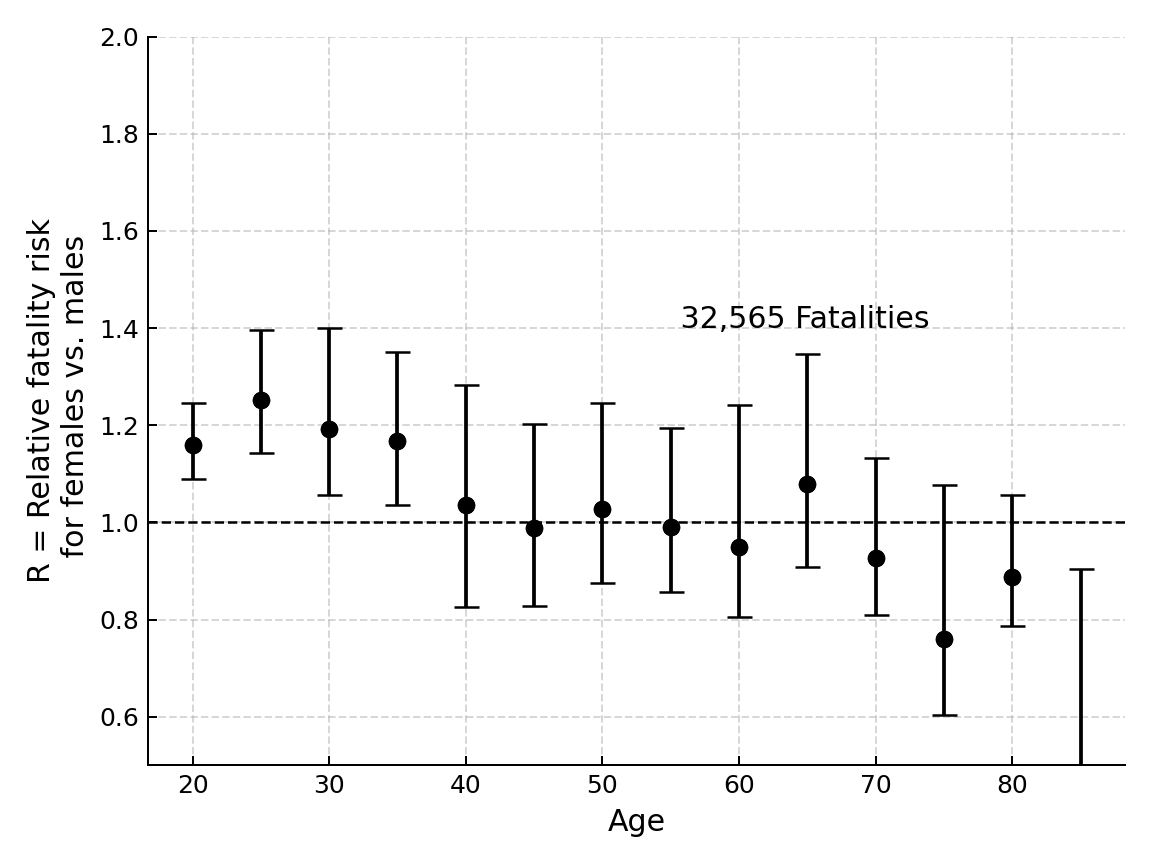


**Figure E. Relative fatality risk, females vs. males, passenger car fatalities, three or more car crashes, 1975-2020.**

**
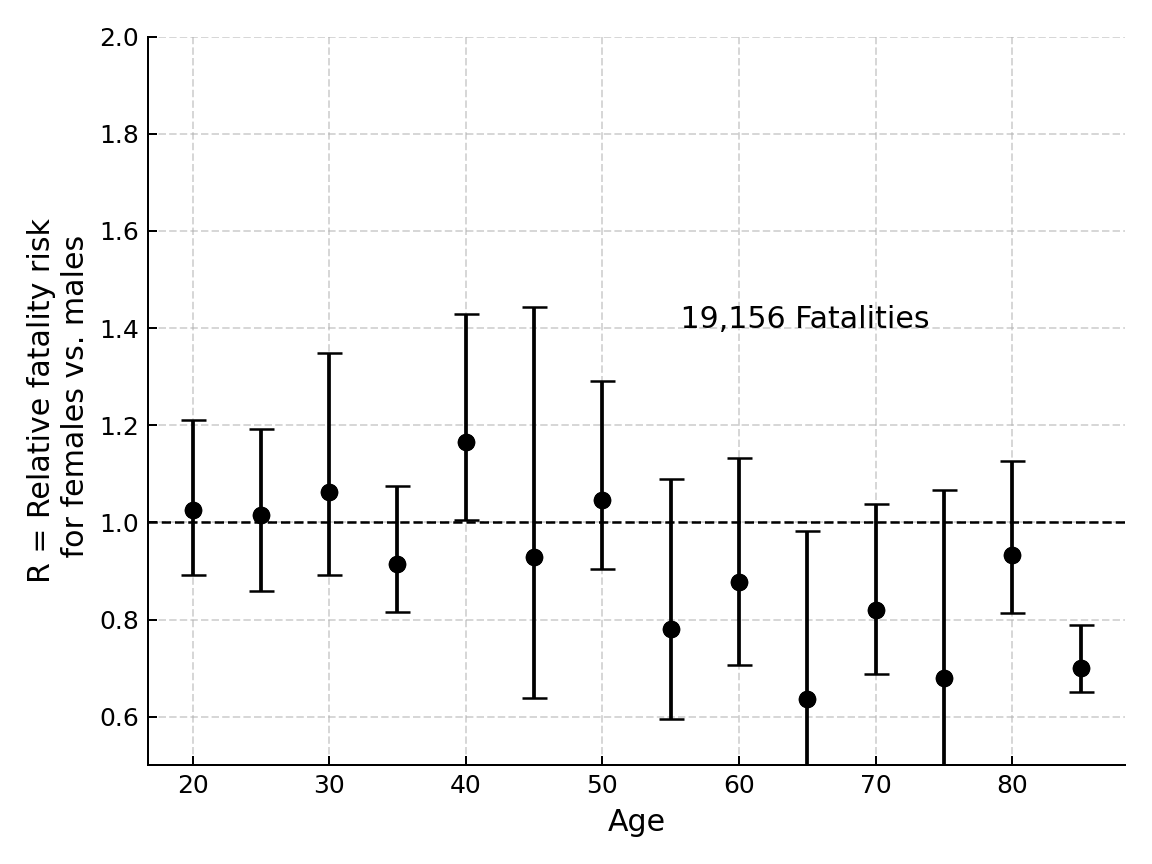
**

**Figure F. Relative fatality risk, females vs. males, light truck fatalities, three or more car crashes, 1975-2020.**

**
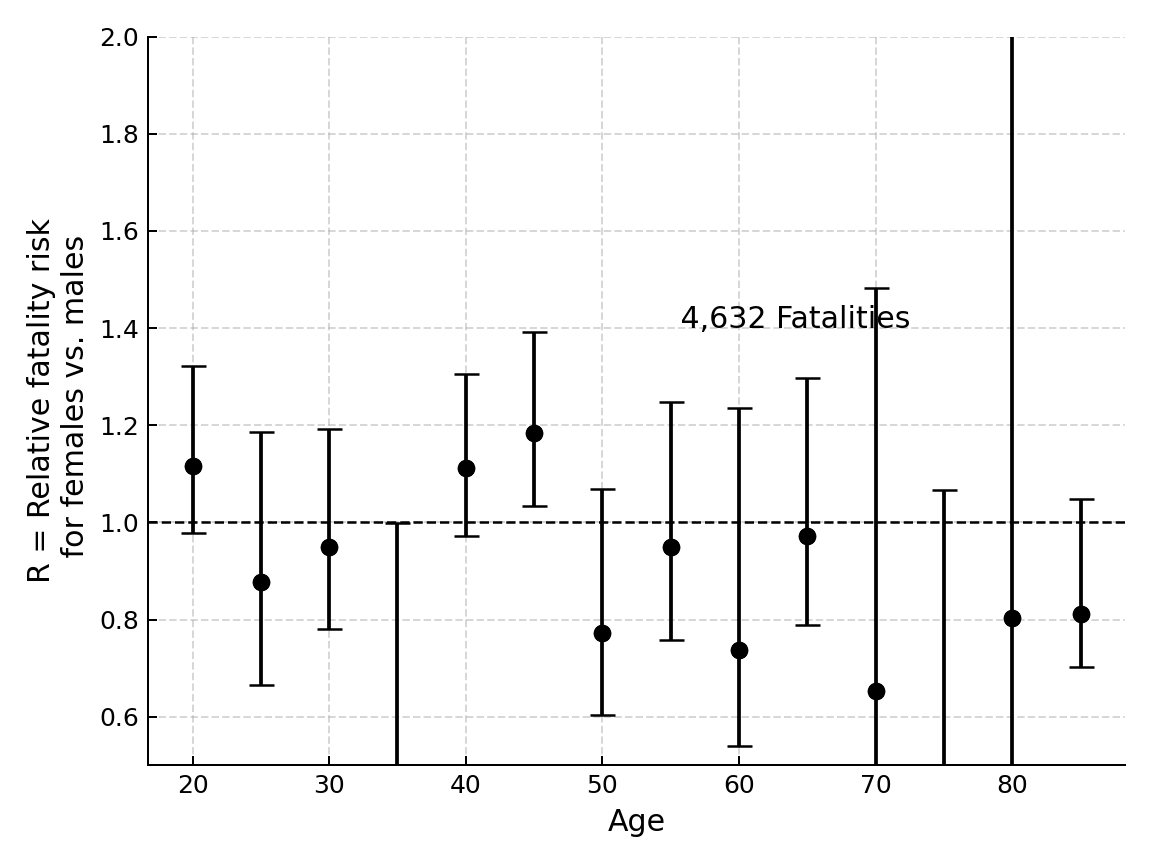
**

**Figure G. Relative fatality risk, females vs. males, passenger car fatalities 1975-2020, frontal impacts only**

Relative fatality risk for female occupants compared to males (*n* = 92,007) under matched airbag deployment conditions in front crashes only. Frontal crashes are defined as crashes between the 11 o’clock position and the 1 o’clock position, as per the FARS Analytical User Manual.


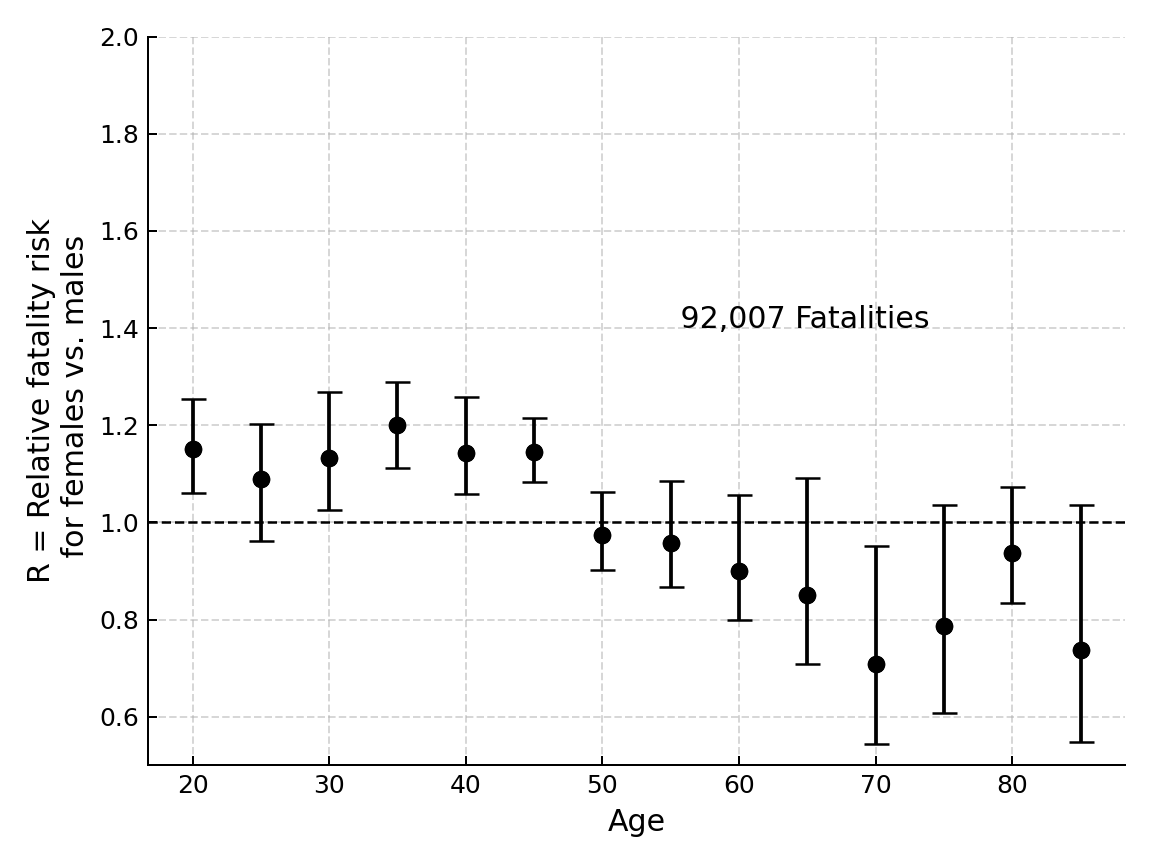


**Figure H. Relative fatality risk, females vs. males, passenger car fatalities 1975-2020, left side impacts only**

Relative fatality risk for all female occupants…


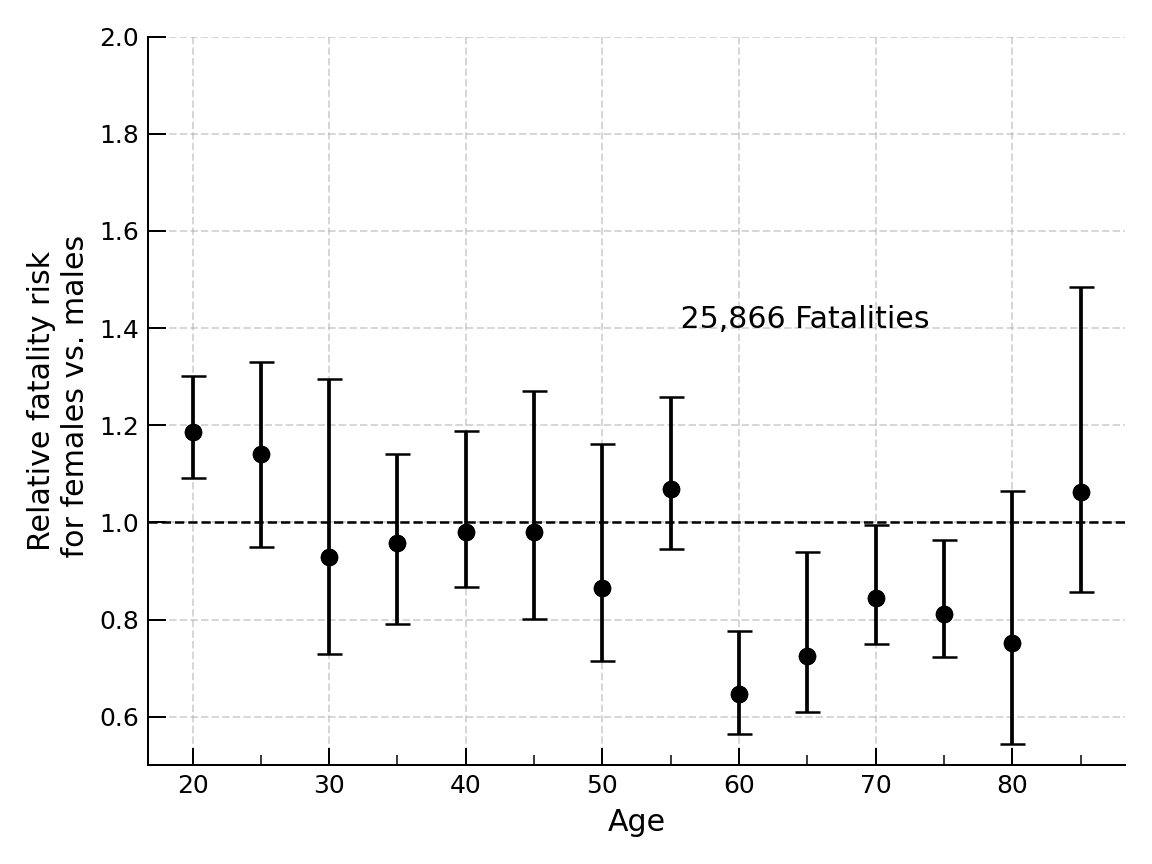


**Figure I. Relative fatality risk, females vs. males, passenger car fatalities 1975-2020, right side impacts only**

Relative fatality risk for all female occupants…

**
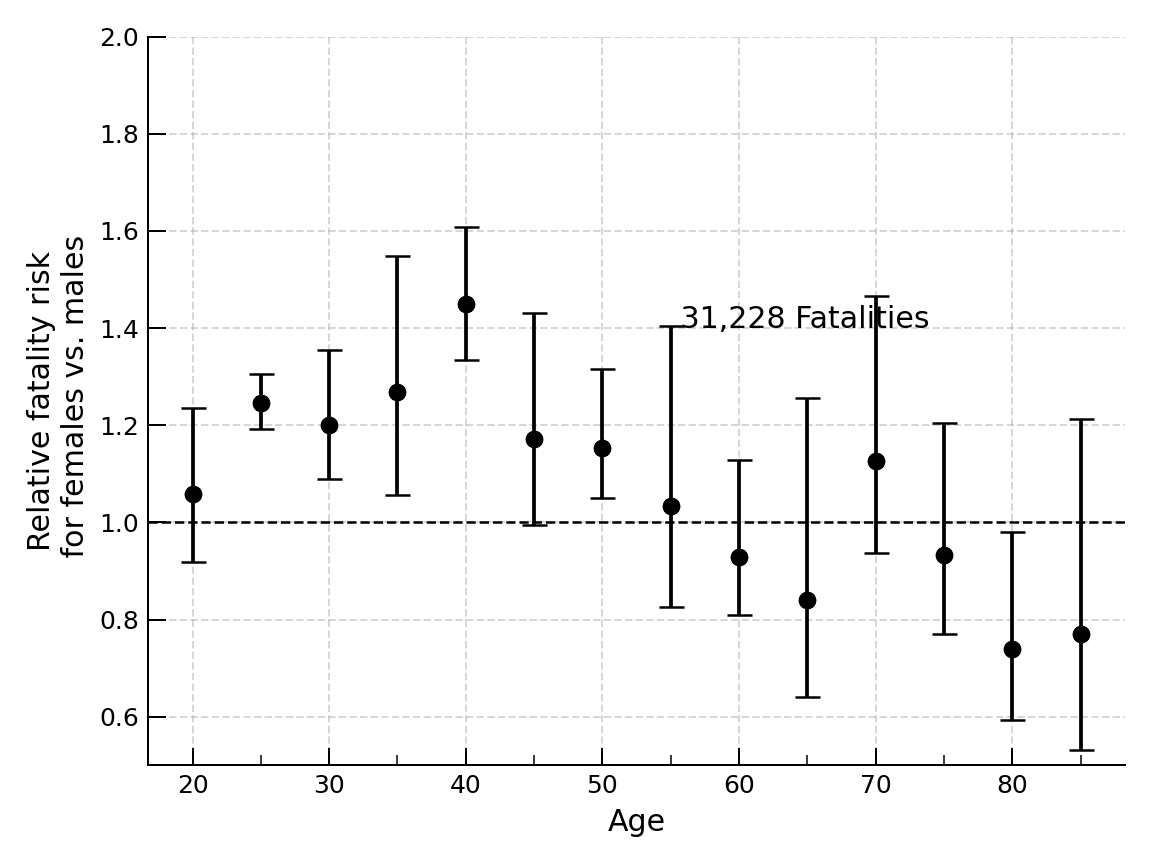
**

**Figure J. Distribution of number of occupants with male and female drivers, 1975-2020.** There is no statistical difference in the distribution of the number of passengers given male or female drivers.


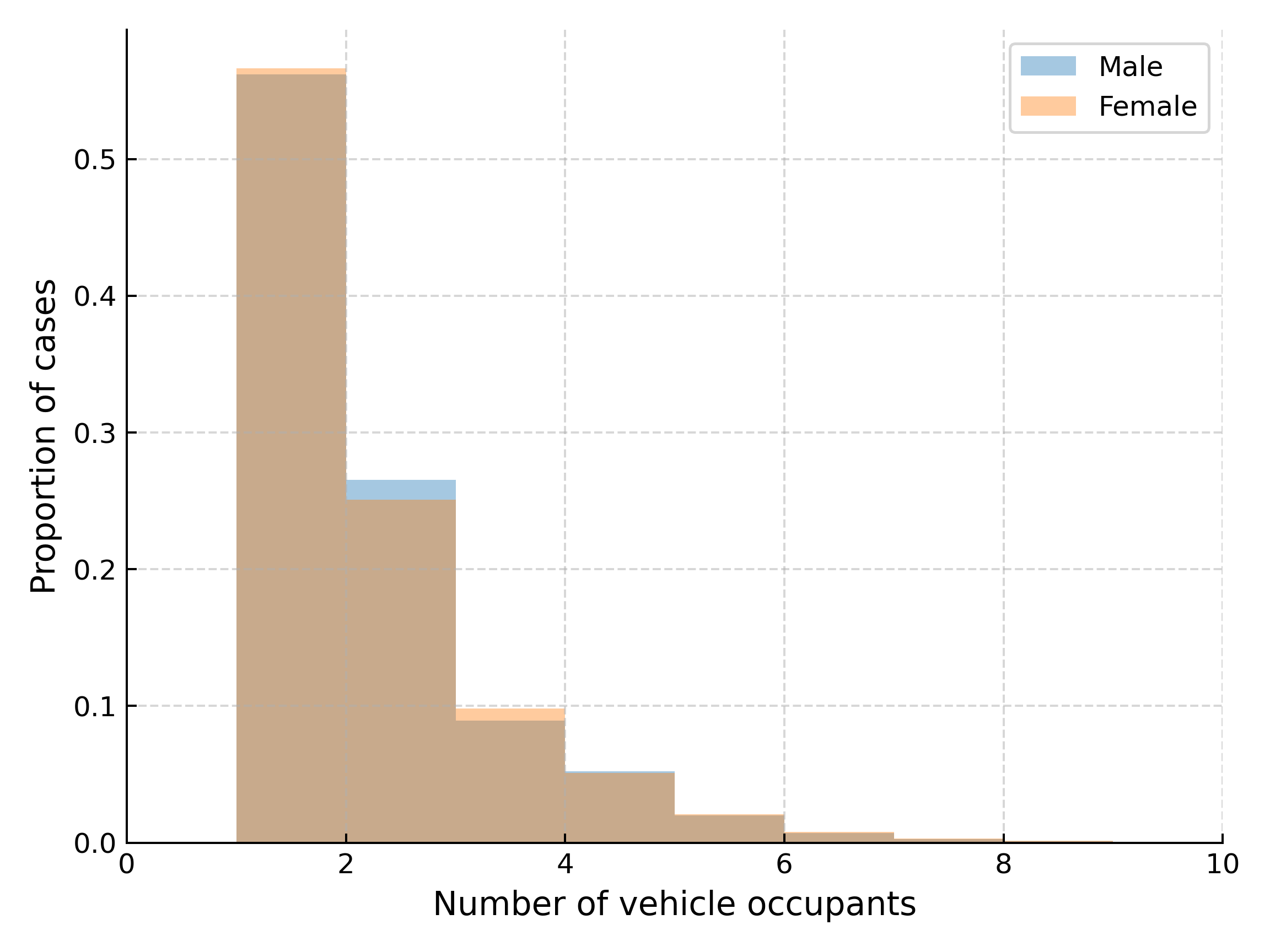


**Figure K. Proportion of cases by number of vehicles involved, for male and female drivers, 1975-2020.**


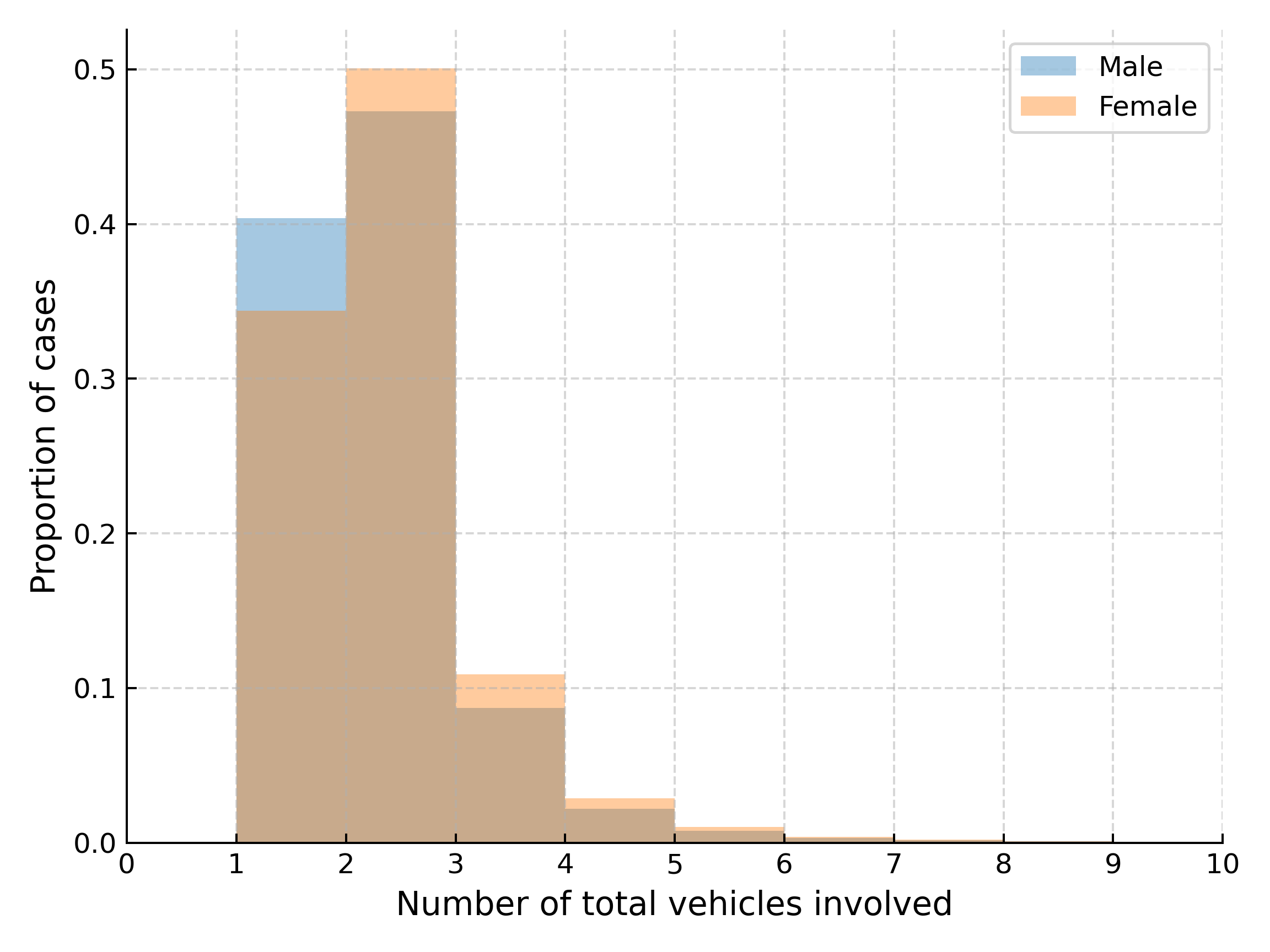

Supplement: S1 Appendix — (DOCX) [file pone.0297211.s001.docx]
